# Short-term outcomes depending on type of oesophagojejunostomy in laparoscopic total gastrectomy for gastric cancer: retrospective study based on a Korean Nationwide Survey for Gastric Cancer in 2019

**DOI:** 10.1093/bjsopen/zrae129

**Published:** 2024-11-01

**Authors:** Gun Kang, Jiyeong Kim, Ju-Hee Lee

**Affiliations:** Department of Surgery, Hanyang University Hospital, Seoul, Republic of Korea; Department of Pre-Medicine, College of Medicine, and Biostatistics Laboratory, Medical Research Collaborating Center (MRCC), Hanyang University, Seoul, Republic ofKorea; Department of Surgery, Hanyang University Hospital, Seoul, Republic of Korea; Department of Surgery, Hanyang University, College of Medicine, Seoul, Republic of Korea

## Abstract

**Background:**

The study aimed to assess postoperative complication rates of different oesophagojejunostomy (EJ) techniques used in laparoscopic total gastrectomy for gastric cancer.

**Methods:**

A total of 1155 patients who underwent laparoscopic total gastrectomy were retrospectively selected from the data obtained from the Korean Nationwide Survey for gastric cancer in 2019. Morbidity rate was compared between patients who received intracorporeal or extracorporeal EJ using linear or circular staplers during laparoscopic total gastrectomy. The variables of the groups were balanced using the inverse probability of treatment weighting.

**Results:**

Seven hundred and seventy-three patients received intracorporeal EJ using a linear stapler (IL), 137 received intracorporeal EJ using a circular stapler (IC), 134 received extracorporeal EJ using a linear stapler (EL) and 111 received extracorporeal EJ using a circular stapler (EC). The overall complication rates were lower in the extracorporeal group (EL: 13.4% *versus* EC: 12.6%) compared to the intracorporeal group (IL: 22.6% *versus* IC: 17.5%) (*P* = 0.006). Fewer major complications were observed in the extracorporeal group (EL: 1.4% *versus* EC: 1.8%) compared to the intracorporeal group (IL: 9.4% *versus* IC: 7.3%) (*P* = 0.004). There was no significant difference in EJ-related complications between the groups (*P* = 0.418 in EJ leakage and *P* = 0.474 in EJ stricture). Multivariable analysis showed that the IL method correlated with more overall and major complications than the extracorporeal method.

**Conclusion:**

The results of this study suggest that despite its widespread use, the IL method is a challenging procedure with higher complication rates than the extracorporeal method. Further high-quality studies are required to confirm the results.

## Teaser

The results of our study suggest that the intracorporeal oesophagojejunostomy using linear staplers method is a difficult procedure with high complication rates when compared with other techniques. Although this study utilized previously collected data, which limited our ability to confirm certain factors, its significance lies in the inclusion of a sufficient number of patients and efforts to minimize bias using inverse probability of treatment weighting.

## Introduction

Gastric cancer remains the fifth most common cancer and the third leading cause of cancer-related deaths worldwide^1^. Laparoscopic surgery has provided an alternative approach for treating gastric cancer. With the development of laparoscopic surgical techniques, the scope of laparoscopic gastrectomy is gradually expanding. Although laparoscopic total gastrectomy (LTG) is increasingly being performed^2^, oesophagojejunostomy (EJ) is still considered a difficult procedure. Globally, surgeons seem to be struggling with establishing the proper method of EJ after LTG^3–9^.

In total open gastrectomy, EJ with a circular stapler is the standard technique. Indeed, the authors prefer this method, even in laparoscopic surgery (intracorporeally), owing to previous research results^10^. However, the implementation of intracorporeal EJ with a circular stapler (IC) seems to be related to anastomosis stenosis according to some studies^11^. Therefore, further studies are needed to determine the optimal anastomosis method.

According to the published nationwide survey data of the Korean Gastric Cancer Association (KGCA), LTG was performed in 1211 patients in 68 hospitals in 2019^2^. In most cases, Roux-en-Y EJ was performed as a reconstruction, but various EJ techniques were performed depending on the surgical approach used (intracorporeal or extracorporeal) and whether the stapler used was circular or linear. The purpose of this study was to compare complications and short-term results of each anastomosis technique and to propose an appropriate anastomosis method after LTG using a large-scale nationwide data set.

## Materials and methods

### Patients and data extraction

Patients (*n* = 1212) who underwent LTG in 2019 were selected from a nationwide survey database of the KGCA. The KGCA has been conducting nationwide retrospective surveys for gastric cancer every 5 years since 1995 and the results have been published regularly^2,12,13^. All institutions registered with the KGCA were invited to participate in this survey *via* email and chose to participate voluntarily. Designated representatives of the institutions were responsible for gathering and submitting data from their respective institutions, as well as correcting any inaccuracies or omissions in the data. The Information Committee of the KGCA was tasked with reviewing the collected data and identifying any inaccuracies or omissions. The survey comprised 54 questions, including those relating to patient demographics, past medical history, pathological findings, operative methods and surgical outcomes. Patient demographics included age, sex, hospital volume, BMI, ASA physical status classification, history of previous abdominal surgery or gastrectomy, and co-morbidities. Co-morbidity variables included diabetes mellitus, hypertension, liver disease, tuberculosis, heart disease, chronic renal failure, chronic obstructive pulmonary disease, cerebrovascular accident and others. The number of co-morbidities was categorized as 0–1 or 2 or more.

Postoperative complications were defined as any complication that occurred within 30 days of surgery. Postoperative complications were categorized into 14 types, including anastomotic leakage, anastomotic stricture, duodenal stump leakage, intra-abdominal bleeding, luminal bleeding, pancreatic fistula, intra-abdominal abscess, fluid collection, wound problem, mechanical ileus, pneumonia, cerebrovascular accident, cardiac disease and others, and the severity of postoperative complications was determined using the Clavien–Dindo classification^14^. Postoperative mortality was defined as death within 30 days of surgery or during hospitalization.

Patients (*n* = 35) with a history of neoadjuvant chemotherapy for gastric cancer and those who received a handsewn EJ procedure (*n* = 3) were excluded. A further 18 patients whose data on the extent of lymph node dissection and stages were unavailable were also excluded given that these factors were used for inverse probability of treatment weighting (IPTW) to balance patients’ baseline characteristics between study groups. Of the remaining 1155 patients, 773 were treated using an intracorporeal EJ with a linear stapler (IL), 137 using an intracorporeal EJ with a circular stapler (IC), 134 using an extracorporeal EJ with a linear stapler (EL), and 111 using an extracorporeal EJ with a circular stapler (EC). This study was approved by the Institutional Review Board of Hanyang University Hospital (IRB no. 2023-09-012).

### Statistical analysis

Statistical analyses were performed using SAS 9.4 (SAS Institute Inc., Cary, NC, USA) and R version 4.2.3 (R Foundation for Statistical Computing, Vienna, Austria). IPTW using propensity score–calculated weights was used to minimize the effect on the difference in confounding factors between the two groups and balance age, sex, extent of lymph node dissection and stages between study groups. All values are expressed as the mean(s.d.) or frequency (percentage). The χ^2^ test, Fisher's exact test and one-way analysis of variance were used for comparisons between the groups. *P* ≤ 0.05 was considered statistically significant. A logistic regression model was used for multivariable analysis to identify independent risk factors for complications, and odds ratios with 95% confidence intervals were calculated before and after IPTW. For the multivariable analysis, only variables with *P* ≤ 0.05 from the univariable analysis were included.

## Results

### Patient demographics and postoperative outcomes

The baseline characteristics were compared between the IL, IC, EL and EC groups before and after weighting using the standardized differences approach, and values that were ±0.1 of the standardized differences were denoted as well-balanced between the two groups (*Table 1*). Before adjustment, the mean age, hospital volume (<100 cases/year *versus* ≥100 cases/year), presence of previous abdominal operation history, surgical intent (curative *versus* palliative), extent of lymph node dissection and type of omentectomy (partial *versus* total) were statistically different between the groups. After IPTW, the hospital volume, presence of previous abdominal operation history and performance of combined surgery remained significantly different between the study groups.

**Table 1:** Clinicopathological characteristics between study groups (*n* = 1155)

*Character*	Unadjusted						IPTW	
	Intracorporeal (n = 910)		Extracorporeal (n = 245)		P	Standardized difference	P	Standardized difference
	IL (n = 773)	IC (n = 137)	EL (n = 134)	EC (n = 111)				
Age (years), mean(s.d.)	63.29(11.77)	64.77(10.98)	64.10(11.06)	60.77(11.67)	0.045*	0.187	0.725*	0.036
**Sex** Male Female	549 224	94 43	103 31	75 36	0.354†	0.113	0.927†	0.037
**Hospital volume**								
** **<100 cases	78 (10.1)	5 (3.7)	3 (2.2)	26 (23.4)	<0.001†	0.383	<0.001†	0.403
≥100 cases	695 (89.9)	132 (96.4)	131 (97.8)	85 (76.6)				
BMI (kg/m^2^), mean(s.d.)	24.03(3.36)	23.58(2.93)	23.85(3.15)	24.23(3.16)	0.383*	0.113	0.789*	0.098
**ASA class**					<0.001†	0.236	0.005†	0.226
0–2	631 (83.3)	121 (88.3)	95 (70.9)	96 (86.5)				
≥3	127 (16.8)	16 (11.7)	39 (29.1)	15 (13.5)				
**Co-morbidities**					0.125†	0.161	0.051†	0.198
0–1	538 (71.7)	53 (60.9)	83 (75.2)	78 (73.6)				
≥2	212 (28.3)	34 (39.1)	27 (24.8)	28 (26.4)				
**Presence of previous operation history**					0.030†	0.212	0.013†	0.232
No	165 (21.9)	17 (19.5)	30 (27.5)	13 (11.7)				
Yes	590 (78.2)	70 (80.5)	79 (72.5)	98 (88.3)				
**Previous gastrectomy**					0.221‡	0.111	0.106†	0.129
No	724 (95.9)	85 (97.7)	107 (98.2)	110 (99.1)				
Yes	31 (4.1)	2 (2.3)	2 (1.8)	1 (0.9)				
**Surgical intent**					<0.001‡	0.141	0.054†	0.371
Curative	753 (97.4)	136 (99.3)	134 (100.0)	108 (100.0)				
Palliative	20 (2.6)	1 (0.7)	–	–				
**Combined operation**					0.106†	0.137	0.035†	0.161
No	695 (90.2)	125 (91.2)	118 (88.1)	92 (82.9)				
Yes	76 (9.8)	12 (8.8)	16 (11.9)	19 (17.2)				
**Lymph node dissection**					<0.001†	0.289	0.999†	0.009
<D2	234 (30.3)	62 (45.3)	76 (56.7)	45 (40.5)				
≥D2	539 (69.8)	75 (54.7)	58 (43.3)	66 (59.5)				
**Omentectomy**					0.048†	0.121	0.322†	0.082
** **Partial	461 (64.2)	65 (74.7)	95 (73.1)	79 (71.2)				
** **Total	257 (35.8)	22 (25.3)	35 (26.9)	32 (28.8)				
**Stage**					0.136†	0.233	1.000†	0.038
** **I	133 (17.2)	26 (19.0)	34 (25.4)	15 (13.5)				
** **II	474 (61.3)	93 (67.9)	75 (56.0)	77 (69.4)				
** **III	145 (18.8)	15 (11.0)	23 (17.2)	16 (14.4)				
** **IV	21 (2.7)	3 (2.2)	2 (1.5)	3 (2.7)				
Overall complication	175 (22.6)	24 (17.5)	18 (13.4)	14 (12.6)	0.010†	0.151	0.006†	0.176
Major complication (CD grade > 3)	72 (9.4)	10 (7.3)	2 (1.5)	2 (1.8)	0.001†	0.223	0.004†	0.22
**EJ related complications**	43 (5.6)	5 (3.6)	4 (3.0)	4 (3.6)	0.447†	0.223	0.614†	0.053
EJ leakage	33 (4.3)	3 (2.2)	4 (3.0)	2 (1.8)	0.527‡	0.081	0.418†	0.097
EJ stricture	10 (1.3)	2 (1.5)	–	2 (1.8)	0.519‡	0.101	0.474†	0.113
Mortality	9 (1.2)	–	–	–	0.027‡	0.078	0.244†	0.076
No. of harvested lymph nodes	42.74 (22.81)	45.15 (20.09)	41.77 (23.75)	48.50 (24.64)	0.053*	0.163	0.005*	0.136
Operation time (min), mean(s.d.)	212.21 (98.84)	131.58 (140.87)	252.40 (70.95)	162.82 (138.42)	<0.001*	0.61	0.139*	0.521
EBL (mL), mean(s.d.)	133.12 (182.43)	88.60 (103.21)	150.19 (128.51)	114.73 (113.64)	0.040*	0.265	0.975*	0.212
Hospital stay (days), mean(s.d.)	11.73 (12.95)	10.83 (10.25)	10.52(9.57)	10.24 (6.63)	0.441*	0.077	0.314*	0.068

Values are *n* (%) unless otherwise indicated. CD, Clavien–Dindo; EBL, estimated blood loss; EC, extracorporeal oesophagojejunostomy with a circular stapler; EJ, oesophagojejunostomy; EL, extracorporeal oesophagojejunostomy with linear staplers; IC, intracorporeal oesophagojejunostomy with a circular stapler; IL, intracorporeal oesophagojejunostomy with linear staplers; IPTW, inverse probability of treatment weighting; *ANOVA; †chi-square test; ‡Fisher's exact test.

The mean(s.d.) number of harvested lymph nodes was >40 in all study groups (IL: 42.74(22.81), IC: 45.15(20.09), EL: 41.77(23.75), EC: 48.50(24.64); *P* = 0.053 and 0.005 before and after IPTW respectively). The mean operating time (*P* < 0.001) and estimated blood loss (*P* = 0.04) were significantly different before IPTW, although the significance was lost after IPTW (*P* = 0.139 and 0.975 respectively). Additionally, there was no significant difference in the postoperative hospital stay between the study groups before and after IPTW (mean(s.d.) number of days 11.73(12.95) *versus* 10.83(10.25) *versus* 10.52(9.57) *versus* 10.24(6.63) in IL *versus* IC *versus* EL *versus* EC groups; *P* = 0.5441 and 0.314 before and after IPTW respectively).

### Postoperative complications

The overall complication rates were significantly different between the groups before and after IPTW (IL: 22.6% *versus* IC: 17.5% *versus* EL: 13.4% *versus* EC: 12.6%; *P* = 0.01 and 0.006 before and after IPTW respectively). Major complication (>grade IIIa) rates were also significantly different (IL: 9.4% *versus* IC: 7.3% *versus* EL: 1.5% *versus* EC: 1.8%; *P* = 0.001 and 0.004 before and after IPTW respectively). However, EJ-related complications (EJ leakage and stricture) were not significantly different between the groups before and after IPTW (5.6% *versus* 3.6% *versus* 3.0% *versus* 3.6% in IL *versus* IC *versus* EL *versus* EC groups; *P* = 0.447 and 0.614 before and after IPTW respectively). The incidence of EJ leakage did not differ significantly between the groups before and after IPTW (4.3% *versus* 2.2% *versus* 3.0% *versus* 1.8% in IL *versus* IC *versus* EL *versus* EC groups; *P* = 0.527 and 0.418 before and after IPTW respectively). With regard to EJ stricture rates, there were no significant differences between groups before and after IPTW (1.3% *versus* 1.5% *versus* 0% *versus* 1.8% in IL *versus* IC *versus* EL *versus* EC groups; *P* = 0.519 and 0.474 before and after IPTW respectively). Nine patients in the IL group died during hospitalization. After IPTW, there was no significant difference in mortality rates between the study groups (*P* = 0.027 and 0.244 before and after IPTW respectively).

A complete list of the observed morbidities for all patients is shown in *Table S1*. Grade II complications were the most common complications in all study groups, although there was no significant difference in the incidence of grade I, II, IIIa, IIIb, IVa and V complications between the study groups after IPTW (*P* = 0.845 in grade I, *P* = 0.599 in grade II, *P* = 0.061 in grade IIIa, *P* = 0.141 in grade IIIb, *P* = 0.129 in grade IVa, and *P* = 0.824 in grade V; *Table 2*).

**Table 2:** Risk factors related to morbidity in patients who underwent laparoscopic total gastrectomy

Variables	No. patients (*n* = 1155)	Overall complications		Major complications		EJ complications	
		*n* (%)	*P*	*n* (%)	*P*	*n* (%)	*P*
**Age (years)**			<0.001*		<0.001*		0.516*
≤70	768	130 (16.9)		43 (5.6)		35 (4.6)	
>70	387	101 (26.1)		43 (11.2)		21 (5.4)	
**Sex**			0.206*		0.013*		0.205*
Male	821	172 (21.0)		71 (8.7)		44 (5.4)	
Female	334	59 (17.57)		15 (4.5)		12 (3.6)	
**BMI (kg/m^2^)**			0.233*		0.388*		0.461*
<25	734	139 (19.0)		51 (7.0)		33 (4.5)	
≥25	421	92 (21.9)		35 (8.4)		23 (5.5)	
**Hospital volume**			0.008*		0.029*		0.034*
<100 cases	112	33 (29.5)		14 (12.7)		10 (8.9)	
≥100 cases	1043	198 (19.0)		72 (7.0)		46 (4.4)	
**Presence of co-morbidities**			0.042*		0.313*		0.936*
0–1	751	147 (19.6)		54 (7.2)		39 (5.2)	
≥2	301	76 (25.3)		27 (9.1)		16 (5.3)	
**Combined operation**			0.130*		0.488*		0.381*
None	1030	200 (19.4)		75 (7.3)		52 (5.1)	
Yes	123	31 (25.2)		11 (9.1)		4 (3.3)	
**Type of reconstruction**			0.010*		0.001*		0.447*
IL	773	175 (22.6)		72 (9.4)		43 (5.6)	
IC	137	24 (17.5)		10 (7.3)		5 (3.7)	
EL	134	18 (13.4)		2 (1.5)		4 (3.0)	
EC	111	14 (12.6)		2 (1.8)		4 (3.6)	
**Previous abdominal surgery**			0.030*		0.617*		0.426*
None	837	164 (20.0)		62 (7.5)		41 (4.9)	
Yes	225	59 (26.2)		19 (8.5)		14 (6.2)	
**Lymph node dissection**			0.603*		0.177*		0.824*
<D2	429	80 (19.2)		25 (6.1)		21 (5.0)	
D2	742	151 (20.5)		61 (8.3)		35 (4.7)	
**Surgical intent**			0.282†		1.000†		0.620†
Curative	1131	229 (20.3)		85 (7.6)		56 (5.0)	
Palliative	21	2 (9.5)		1 (4.8)		-	
**Omentectomy**			0.162*		0.453*		0.073*
Partial	700	147 (21.0)		22 (6.4)		11 (3.2)	
Total	346	60 (17.3)		53 (7.7)		40 (5.7)	
**Operation time (min)**			<0.001*		0.025*		0.035*
≤240	711	120 (16.9)		43 (6.1)		27 (3.8)	
>240	444	111 (25.0)		43 (9.7)		29 (6.3)	
**Stages**			<0.001*		0.051*		0.478*
I	208	48 (23.1)		45 (6.3)		13 (6.3)	
II	719	122 (17.0)		20 (9.6)		33 (4.6)	
III	199	58 (29.2)		21 (10.6)		10 (5.0)	
IV	29	3 (10.3)		–		–	

EC, extracorporeal circular stapler; EJ, oesophagojejunostomy; EL, extracorporeal linear stapler; IC, intracorporeal circular stapler; IL, intracorporeal linear stapler; *chi-square test, †Fisher's exact test.

### Risk factors associated with postoperative morbidity

Risk factors associated with overall complications, major complications and EJ-related complications were evaluated. Variables associated with the occurrence of overall complications in univariate analysis were as follows: age >70 years (*P* < 0.001), hospital volume <100 cases (*P* = 0.008), co-morbidity ≥2 (*P* = 0.042), type of reconstruction (*P* = 0.01), history of previous abdominal surgery (*P* = 0.03), operation time ≥240 min (*P* < 0.001) and stages (*P* < 0.001) (*Table 2*). Multivariable analysis showed that age >70 years, type of reconstruction (IL group *versus* EL or EC group), history of previous abdominal surgery and longer operation time (>240 min) were predictable risk factors for the occurrence of overall complications both before and after IPTW (*Table 3*).

**Table 3:** Risk factors related to overall complications in patients who underwent laparoscopic total gastrectomy: multivariable analysis

Variables	Before IPTW			After IPTW		
	OR	95% c.i.	*P*	OR	95% c.i.	*P*
Age (>70 *versus* ≤70 years)	1.653	1.202,2.273	0.002	1.625	1.182,2.233	0.003
Hospital volume (<100 *versus* ≥100)	1.42	0.890,2.268	0.142	1.496	0.945,2.367	0.086
Presence of co-morbidity (≥2 *versus* 0–1)	1.18	0.844,1.650	0.332	1.175	0.840,1.643	0.346
**Type of reconstruction**						
IC *versus* IL	0.658	0.365,1.187	0.164	0.685	0.387,1.211	0.193
EL *versus* IL	0.551	0.317,0.959	0.035	0.495	0.284,0.864	0.013
EC *versus* IL	0.511	0.278,0.939	0.031	0.407	0.216,0.766	0.005
Previous abdominal surgery (yes *versus* no)	1.437	1.009,2.047	0.045	1.459	1.027,2.073	0.035
Operation time (>240 *versus* ≤240 min)	1.768	1.281,2.440	0.001	1.781	1.290,2.458	0.001
**Stages**						
II *versus* I	0.705	0.471,1.056	0.09	0.718	0.481,1.073	0.106
III *versus* I	1.164	0.726,1.867	0.528	1.236	0.772,1.981	0.378
IV *versus* I	0.322	0.091,1.139	0.079	0.273	0.068,1.097	0.067

EC, extracorporeal oesophagojejunostomy with a circular stapler; EL, extracorporeal oesophagojejunostomy with linear staplers; IC, intracorporeal oesophagojejunostomy with a circular stapler; IL, intracorporeal oesophagojejunostomy with linear staplers; IPTW, inverse probability of treatment weighting.

Next, variables associated with major complications were analysed. Univariate analysis showed that age >70 years (*P* < 0.001), male sex (*P* = 0.013), hospital volume <100 cases (*P* = 0.029), type of reconstruction (*P* = 0.001) and operation time ≥240 min (*P* = 0.025) were associated with the occurrence of major complications (*Table 2*). Before IPTW, age >70 years, male sex, type of reconstruction (IL group *versus* EL or EC group) and longer operation time (>240 min) were identified as independent risk factors associated with major complications in multivariable analysis (*Table 4*). After IPTW, the male factor lost statistical significance.

**Table 4:** Risk factors related to major complications in patients who underwent laparoscopic total gastrectomy: multivariable analysis

Variables	Before IPTW			After IPTW		
	OR	95% c.i.	*P*	OR	95% c.i.	*P*
Age (>70 *versus* ≤70 years)	1.927	1.226,3.030	0.005	1.832	1.168,2.871	0.008
Sex (male *versus* female)	1.89	1.056,3.382	0.032	1.691	0.962,2.971	0.068
Hospital volume (<100 *versus* ≥100)	1.699	0.892,3.233	0.107	1.642	0.868,3.106	0.127
**Type of reconstruction**						
IC *versus* IL	0.755	0.375,1.520	0.431	0.846	0.431,1.662	0.627
EL *versus* IL	0.123	0.029,0.513	0.004	0.24	0.080,0.718	0.011
EC *versus* IL	0.174	0.042,0.728	0.017	0.128	0.026,0.629	0.011
Operation time (>240 *versus* ≤240 min)	1.637	1.035,2.590	0.035	1.661	1.053,2.620	0.029

EC, extracorporeal oesophagojejunostomy with a circular stapler; EL, extracorporeal oesophagojejunostomy with linear staplers; IC, intracorporeal oesophagojejunostomy with a circular stapler; IL, intracorporeal oesophagojejunostomy with linear staplers; IPTW, inverse probability of treatment weighting.

In univariable analysis, the occurrence of EJ-related complications was related to hospital volume <100 cases/year (*P* = 0.034) and operation time ≥240 min (*P* = 0.035) (*Table 2*). In multivariable analysis after IPTW, operation time ≥240 min was the only risk factor associated with EJ-related complications (*Table 5*). The type of reconstruction was not a predictor for EJ-related complications in univariable and multivariable analyses.

**Table 5:** Risk factors related to oesophagojejunostomy-related complications in patients who underwent laparoscopic total gastrectomy: multivariable analysis

Variables	Before IPTW			After IPTW		
	OR	95% c.i.	*P*	OR	95% c.i.	*P*
Hospital volume (<100 *versus* ≥100)	1.893	0.916,3.908	0.085	1.786	0.888,3.594	0.104
Operation time (>240 *versus* ≤240 min)	1.654	0.958,2.857	0.071	1.766	1.035,3.014	0.037

IPTW, inverse probability of treatment weighting.

## Discussion

In this study, the occurrences of overall, major and EJ-related complications were not significantly different between the IL and IC groups, or between the IC and extracorporeal anastomosis (EC or EL) groups. However, the incidence of overall and major complications was significantly higher in the IL group than in the extracorporeal anastomosis (EC or EL) groups. These results indicate that extracorporeal anastomosis seems to be safer and more feasible compared to IL anastomosis. Nevertheless, caution is needed in interpretation when considering the limitations of the data and changes in techniques over time. Moreover, no significant differences were observed in EJ-related complications among all types of anastomoses after LTG. Determining the appropriate method will require further discussion.

In the early period when laparoscopic surgery was introduced, extracorporeal anastomosis was widely performed. However, extracorporeal anastomosis is conducted in a limited working space with restricted vision, thus making the procedure difficult, especially in obese patients. The intracorporeal anastomosis method was introduced with the hope of overcoming the difficulty of reconstruction, especially in obese patients. For distal gastrectomy, some studies have suggested that intracorporeal anastomosis is superior in terms of postoperative recovery^15–17^, whereas others regard extracorporeal anastomosis to be better^18,19^. Most studies have reported that the incidence of anastomosis-related complications is not significantly different between the procedures. Therefore, the Korean Laparoendoscopic Gastrointestinal Surgery study group (KLASS) 07 study was initiated to compare the procedures in terms of cosmesis, economics and patient's quality of life. This study is expected to confirm the optimal procedure after laparoscopic distal gastrectomy with regard to various aspects. The optimal anastomosis technique for LTG has also not yet been confirmed. Direct comparison of various methods is difficult because detailed techniques differ depending on individual surgeons, even for the same intracorporeal or extracorporeal anastomosis. A meta-analysis by Chen *et al*. showed that there were no significant differences in the operation time, estimated blood loss, time to first flatus, length of hospital stays, or overall and anastomosis-related complications between intracorporeal and extracorporeal EJ anastomosis^20^. Another recent retrospective large-scale single-centre study from Korea reported that IL can be recommended as an appropriate procedure because of shorter operation time, fewer intraoperative anastomosis events and faster postoperative recovery compared to the EC method, although the complications were similar between the two groups^21^. Based on these research results, the intracorporeal method appears to be the preferred method for both partial and total gastrectomy, as shown in the KGCA-led nationwide survey 2019, which demonstrated a notable increase in the use of the intracorporeal method compared to the previous survey^2^. In the present study, compared to extracorporeal anastomosis, intracorporeal anastomosis (especially the IL method) was found to be associated with increased rates of overall and major complications. Moreover, given the relatively recent multicentre data, this study can be considered more reliable than other studies; however, the lack of important data makes it difficult to confirm the results. The present study lacks data on intraoperative events and surgeons’ learning curves, both of which are closely related to postoperative complications but are difficult to investigate through the surveys. Randomized controlled studies balancing these factors are needed to determine the best technique.

EJ-related complication is the most important factor that determines which anastomosis method a surgeon will perform in total gastrectomy. The rate of EJ leakage after LTG is reported to range from 0.7% to 9.5%^22^. Most studies have shown no significant difference between anastomosis procedures during LTG in terms of EJ leakage^7,9,11,23–25^. However, studies on EJ stenosis, especially between IL and IC methods, have reported slightly different opinions. According to two multicentre studies from Korea and Japan, stenosis of the intracorporeal EJ anastomosis was less frequent with the linear stapling technique than with the circular stapling technique^11,23^. Yang *et al*. suggested that the use of an OrVil™ or a 21 mm circular stapler may be the cause of more frequent EJ stenosis in the IC group^11^. Similarly, Murakami *et al*. speculated that the IL method may create a larger anastomosis than the IC reconstruction method, thereby lowering the incidence of anastomotic stenosis^23^. In contrast, Kang *et al*. reported no significant difference in EJ-related complication rates between the IC and IL methods^25^. They suggested that the reason for the lack of a difference in anastomosis stenosis between the methods is due to the use of a single circular stapling technique as the IC method. Several studies have found that this technique causes significantly lower rates of EJ stenosis compared to the hemi-double-stapling technique, which uses a linear stapler to create the oesophageal stump, causing the stapler line to overlap after the circular stapler is fired^26,27^. In the present study, the rates of EJ-related complications were similar between the groups and the results did not change even when the EJ-related complications were subdivided into EJ leakage and stricture. Considering that patients who had undergone surgery relatively recently were also included, it seems that the IC technique is becoming more stable as the surgical society's experience accumulates. However, it is difficult to make a judgement based on these results alone, given that specific individual procedures were not surveyed. Additionally, another limitation is that EJ-related strictures were only analysed for 30 days after surgery, which was not a sufficient period of time to comprehensively evaluate long-term EJ-related strictures. Tumour location is also an important indicator to consider when deciding the EJ surgical technique. Because the IL method requires a longer oesophageal length for anastomosis, the IC method might be preferred when the tumour is located near or above the gastro-oesophageal junction. This may be one of the reasons why more complications are reported with the IC method than with the IL method in previous studies^7,9,11,23^. However, presence or absence of oesophageal or gastro-oesophageal junction invasion was not separately surveyed in the present study. This is also an important consideration when interpreting the results.

The authors used a propensity score based on IPTW adjustment, which significantly reduces confounding bias and imbalance in covariates and thus potentially offers an estimation of treatment effect similar to that in randomized trials^28^. It assigns weights to each observation based on the probability of receiving the treatment that was actually received, thereby balancing the distribution of covariates between treatment groups. Using the entire cohort of 1155 patients, the IPTW approach increased the study power compared to matched-pairs analysis^29^. The authors selected only variables (age, sex, extent of lymph node dissection and stages) that were strongly associated with the incidence of complications in gastric cancer rather than including all baseline variables. After IPTW adjustment, hospital volume, history of abdominal surgery and performance of combined operation remained as differences between the groups. Although these factors can influence the occurrence of complications, low hospital volume (<100 cases), history of abdominal surgery and performance of combined operation were not most frequent in the IL group, which had the highest rates of overall and major complications. Moreover, in multivariable analysis to further evaluate whether the type of reconstruction influences the complication rate, the IL method remained a significant risk factor.

In conclusion, the authors found that the IL method is a demanding procedure with a significant risk of complications compared to the extracorporeal EJ methods. However, considering that the EJ procedure has been transitioning to an intracorporeal method as investigated in this study, either the IL or IC method can be performed with no significant differences in overall, major and EJ-related complication rates. Although this study utilized previously collected data, which limited the ability to confirm certain factors, its significance lies in the inclusion of a considerable number of patients and efforts to minimize bias using IPTW. A well-designed randomized clinical trial is necessary to confirm the optimal anastomosis method for EJ in LTG.

## Supplementary Material

zrae129_Supplementary_Data

## Data Availability

The data that support the findings of this study are available from the corresponding author upon reasonable request.
