# Supplementary material for: Short-term outcomes depending on type of oesophagojejunostomy in laparoscopic total gastrectomy for gastric cancer: retrospective study based on a Korean Nationwide Survey for Gastric Cancer in 2019
Source: BJS Open. 2024 Nov 1;8(6):zrae129. doi: 10.1093/bjsopen/zrae129 (PMC11528302; doi:10.1093/bjsopen/zrae129)
Supplement: zrae129_Supplementary_Data [file zrae129_supplementary_data.docx]

**Short-term outcomes depending on type of esophagojejunostomy in laparoscopic total gastrectomy for gastric cancer: Retrospective study based on a Korean Nationwide Survey for Gastric Cancer in 2019**

Gun Kang, MD^1^; Jiyeong Kim, PhD^3^; Ju-Hee Lee, MD. PhD^1,2^

^1^Department of Surgery, Hanyang University Hospital, Seoul, Republic of Korea

^2^Department of Surgery, Hanyang University, College of Medicine, Seoul, Republic of Korea

^3^Department of Pre-Medicine, College of Medicine, and Biostatistics Laboratory, Medical Research Collaborating Center (MRCC), Hanyang University, Seoul, Korea

**Corresponding author.**

Ju-Hee Lee, MD, PhD

Department of Surgery, College of Medicine, Hanyang University

222 Wangsimni-ro, Seongdong-gu, Seoul, Republic of Korea

Tel.: +82-2-2290-8451

Fax: +82-504-282-4248

[E-mail: leejuhee79@gmail.com](mailto:E-mail: jhlee7979@hanyan.ac.kr)

ORCID: [https://orcid.org/0000-0003-0298-6275](https://orcid.org/0000-0003-0298-6275?lang=en)

**Supplementary Materials - Index**

| **Supplementary Figures and Tables** |  |
| --- | --- |
| Comparison of postoperative morbidity after laparoscopic total gastrectomy according to type of anastomosis | *page 3* |
|  |  |
|  |  |
|  |  |

**Supplementary Figures and Tables**

| **Suppl. Table 1** Comparison of postoperative morbidity after laparoscopic total gastrectomy according to type of anastomosis | | | | | | | | | |
| --- | --- | --- | --- | --- | --- | --- | --- | --- | --- |
|  | Unadjusted | | | | | |  | IPTW | |
| **Grades** | Intracorporeal (n = 910) | | Extracorporeal (n = 245) | | *p-value* | Standardized difference |  | *p-value* | Standardized difference |
|  | IL (n = 773) | IC  (n = 137) | EL  (n = 134) | EC  (n = 111) |  |  |  |  |  |
| **Grade I** | 17 (2.2 %) | 5 (3.6%) | 4 (3.0%) | 2 (1.8%) | 0.628^b^ | 0.065 |  | 0.845^b^ | 0.059 |
| Anstomosis leakage |  |  | 1 |  |  |  |  |  |  |
| Anastomisis stricture |  | 1 |  |  |  |  |  |  |  |
| Fluid collection | 1 |  |  |  |  |  |  |  |  |
| Wound problem | 2 |  | 1 |  |  |  |  |  |  |
| Ileus | 4 |  | 1 |  |  |  |  |  |  |
| Pneumonia | 3 | 1 | 1 |  |  |  |  |  |  |
| Heart problem | 1 |  |  |  |  |  |  |  |  |
| Chyle leakage | 3 |  |  |  |  |  |  |  |  |
| Urinary tract infection | 1 |  |  |  |  |  |  |  |  |
| Others | 2 | 3 |  | 2 |  |  |  |  |  |
| **Grade II** | 78 (10.1%) | 9 (6.6%) | 12 (9.0%) | 9 (8.1%) | 0.581^a^ | 0.069 |  | 0.599^a^ | 0.046 |
| Anstomosis leakage | 7 |  | 1 | 1 |  |  |  |  |  |
| Anastomisis stricture | 3 |  |  | 2 |  |  |  |  |  |
| Duodenal stump leakage | 6 |  |  |  |  |  |  |  |  |
| Anastomosis bleeding | 4 |  | 1 |  |  |  |  |  |  |
| Fluid collection | 6 | 1 | 3 |  |  |  |  |  |  |
| Ileus | 5 | 3 | 1 |  |  |  |  |  |  |
| Pneumonia | 15 |  | 5 | 4 |  |  |  |  |  |
| Wound problem | 2 | 1 |  | 1 |  |  |  |  |  |
| Pancreatic fistula or pancreatitis | 5 | 1 |  |  |  |  |  |  |  |
| Intraabdominal abscess | 7 | 1 |  |  |  |  |  |  |  |
| Heart problem | 2 |  |  |  |  |  |  |  |  |
| Chyle leakage | 2 |  |  |  |  |  |  |  |  |
| Others | 13 | 2 |  | 1 |  |  |  |  |  |
| **Grade IIIa** | 41 (5.3%) | 9 (6.6%) | 2 (1.5%) | 1 (0.9%) | 0.037^a^ | 0.189 |  | 0.061^a^ | 0.195 |
| Anastomosis leakage | 15 | 3 | 2 |  |  |  |  |  |  |
| Anastomosis stricture | 3 |  |  |  |  |  |  |  |  |
| Duodenal stump leakage | 1 | 2 |  |  |  |  |  |  |  |
| Intraabdominal bleeding | 1 |  |  |  |  |  |  |  |  |
| Anastomosis bleeding | 1 |  |  |  |  |  |  |  |  |
| Pancreatic fistula | 1 |  |  |  |  |  |  |  |  |
| Intraabdominal abscess | 2 |  |  |  |  |  |  |  |  |
| Fluid collection | 2 |  |  |  |  |  |  |  |  |
| Wound problem | 1 | 2 |  |  |  |  |  |  |  |
| Ileus | 2 |  |  |  |  |  |  |  |  |
| Pneumonia | 7 | 1 |  |  |  |  |  |  |  |
| Heart problem | 3 |  |  | 1 |  |  |  |  |  |
| Others | 2 | 1 |  |  |  |  |  |  |  |
| **Grade IIIb** | 15 (1.9%) | 1 (0.7%) | 0 (0%) | 0 (0%) | 0.193^b^ | 0.124 |  | 0.141^b^ | 0.125 |
| Anastomosis leakage | 5 |  |  |  |  |  |  |  |  |
| Anastomosis stricture | 3 | 1 |  |  |  |  |  |  |  |
| Wound problem | 2 |  |  |  |  |  |  |  |  |
| Ileus | 4 |  |  |  |  |  |  |  |  |
| Others | 1 |  |  |  |  |  |  |  |  |
| **Grade IVa** | 14 (1.8%) | 0 (0%) | 0 (0%) | 1 (0.9%) | 0.181^b^ | 0.122 |  | 0.129^b^ | 0.12 |
| Anastomosis leakage | 5 |  |  | 1 |  |  |  |  |  |
| Anastomosis stricture | 1 |  |  |  |  |  |  |  |  |
| Wound problem | 2 |  |  |  |  |  |  |  |  |
| Ileus | 3 |  |  |  |  |  |  |  |  |
| Others | 3 |  |  |  |  |  |  |  |  |
| **Grade V** | 2 (0.3%) | 0 (0%) | 0 (0%) | 0 (0%) | 1.000^b^ | 0.036 |  | 0.824^b^ | 0.035 |
| Duodenal stump leakage | 1 |  |  |  |  |  |  |  |  |
| Intraabdominal bleeding | 1 |  |  |  |  |  |  |  |  |
| **Total** | 175 (22.6%) | 24 (17.5%) | 18 (13.4%) | 14 (12.6%) | 0.010^a^ | 0.151 |  | 0.006^a^ | 0.176 |
| IPTW: Inverse probability of treatment weighting, IL: Intracorporeal esophagojejunostomy with linear staplers, IC: Intracorporeal esophagojejunostomy with a circular stapler, EL: Extracorporeal esophagojejunostomy with linear staplers, EC: Extracorporeal esophagojejunostomy with a circular stapler, a: Chi-square test, b: Fisher’s exact test | | | | | | | | | |
